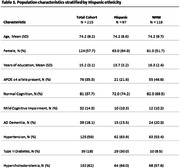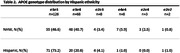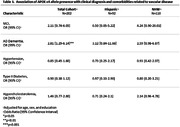# APOE ε4 is associated with risk of clinical Alzheimer’s Disease Dementia independent of Hispanic ethnicity

**DOI:** 10.1002/alz.091765

**Published:** 2025-01-09

**Authors:** Amaya M Seidl, Marco I Boisselier, Ashley LaRoche, Chen‐Pin Wang, Daniel MacCarthy, Jennifer G Del Bosque, Haritha V. Katragadda, Aishwarya N. Patel, Julia J Mathews, Jazmyn A Muhammad, Julie Parker‐Garza, Xianlin Han, Jeremy A. Tanner, Monica Goss, Arash Salardini, Alicia S. Parker, Gabriel A. de Erausquin, A. Campbell Sullivan, Mitzi M. Gonzales, Claudia L Satizabal, Hector A Trevino, Amy Werry, Amy R. Saklad, Gabrielle Hromas, Robin C. Hilsabeck, Shannon Lavigne, Silvia Mejia‐Arango, Stephanie Santiago‐Mejias, Rosa P. Pirela‐Mavarez, Gladys E. Maestre, Sudha Seshadri, Joanne Curran, Tiffany F. Kautz

**Affiliations:** ^1^ Glenn Biggs Institute for Alzheimer’s and Neurodegenerative Diseases, UT Health San Antonio, San Antonio, TX USA; ^2^ Glenn Biggs Institute for Alzheimer’s & Neurodegenerative Diseases, University of Texas Health Sciences Center at San Antonio, San Antonio, TX USA; ^3^ Glenn Biggs Institute for Alzheimer’s & Neurodegenerative Diseases, University of Texas Health Science Center, San Antonio, TX USA; ^4^ Glenn Biggs Institute for Alzheimer’s & Neurodegenerative Diseases, San Antonio, TX USA; ^5^ Department of Population Health Sciences, University of Texas Health Science Center San Antonio, San Antonio, TX USA; ^6^ Glenn Biggs Institute for Alzheimer's & Neurodegenerative Diseases, University of Texas Health Science Center, San Antonio, TX USA; ^7^ Glenn Biggs Institute for Alzheimer’s & Neurodegenerative Diseases, The University of Texas Health Science Center at San Antonio, San Antonio, TX USA; ^8^ University of Texas Health Science Center San Antonio, San Antonio, TX USA; ^9^ University of Texas Health Sciences Center, San Antonio, San Antonio, TX USA; ^10^ Barshop Institute for Longevity and Aging Studies, University of Texas Health Science Center at San Antonio, San Antonio, TX USA; ^11^ Glenn Biggs Institute for Alzheimer’s & Neurodegenerative Diseases, University of Texas Health San Antonio, San Antonio, TX USA; ^12^ Glenn Biggs Institute for Alzheimer’s and Neurodegenerative Diseases, University of Texas Health San Antonio, San Antonio, TX USA; ^13^ Cedars‐Sinai Medical Center, Los Angeles, CA USA; ^14^ Glenn Biggs Institute for Alzheimer’s & Neurodegenerative Diseases, University of Texas Health Science Center at San Antonio, San Antonio, TX USA; ^15^ UT Health San Antonio, San Antonio, TX USA; ^16^ The University of Texas Rio Grande Valley School of Medicine, Harlingen, TX USA; ^17^ The University of Texas Rio Grande Valley School of Medicine, Brownsville, TX USA; ^18^ Department of Neurology, University of Texas Health Sciences Center, San Antonio, TX USA; ^19^ University of Texas Rio Grande Valley, Harlingen, TX USA

## Abstract

**Background:**

The Apolipoprotein E (APOE) ε4 variant is the strongest genetic risk factor for Alzheimer’s disease (AD) and has been thoroughly studied in non‐Hispanic whites (NHW). However, its association with AD among Hispanic individuals is unclear, with existing studies yielding mixed results. Genetics do not entirely explain the likelihood of developing AD. For example, several common vascular contributors are more common in Hispanic adults and have been associated with cognitive impairment and dementia. We sought to characterize the associations between Hispanic ethnicity with APOEε4, cognitive diagnosis, and vascular contributors using data from the South Texas AD Research Center (STAC, *P30AG066546*).

**Method:**

Our study sample was derived from 215 STAC participants (N = 97 Hispanic, N = 118 NHW). Descriptive statistics of demographics and baseline characteristics were calculated for Hispanic and NHW participants (frequency and percentage for categorical variables, mean and standard deviation for continuous variables, **Table 1**). **Table 2** shows APOE4 genotype distribution by ethnicity. Ethnicity‐specific associations between APOE4 and clinical diagnosis were assessed using multivariable logistic regression models, adjusting for covariates (age, sex, education). Multivariable regression models were used to assess the associations between APOE4 with clinical diagnosis adjusting for covariates (**Table 3**). Similar approaches were conducted for the combined sample with ethnicity by APOE4 interaction to test the ethnic differences. All analyses were conducted using SAS 9.4.

**Result:**

We found APOEε4 was associated with AD‐dementia in all participants (OR = 2.81, 95% CI 1.29‐6.14, p = 0.0095). However, we did not find any interaction with ethnicity (in stratified analyses, NHW OR = 2.58, 95% CI 0.97‐6.86, p = 0.06; Hispanic OR = 3.12, 95% CI 0.84‐11.66, p = 0.09). APOEε4 was not significantly associated with MCI, hypertension, hypercholesterolemia, or type II diabetes mellitus, regardless of ethnicity.

**Conclusion:**

Identifying and targeting population‐specific risk factors is crucial to developing effective prevention and treatment approaches for AD in the disproportionately burdened Hispanic population. Our findings support the established association between APOE ε4 and AD risk in both Hispanic and NHW adults. In our STAC cohort, we found no significant relationship between Hispanic ethnicity and APOE ε4. However, our study is limited by the small sample size, which we plan to expand in the future.